# Stability of novel urinary biomarkers used for lupus nephritis

**DOI:** 10.3389/fped.2022.974049

**Published:** 2022-07-29

**Authors:** Ellen M. Cody, James E. Rose, Bin Huang, Tingting Qiu, Hermine I. Brunner, Prasad Devarajan

**Affiliations:** ^1^Division of Nephrology and Hypertension, Cincinnati Children's Hospital Medical Center, Cincinnati, OH, United States; ^2^Division of Biostatics and Epidemiology, Cincinnati Children's Hospital Medical Center, Cincinnati, OH, United States; ^3^School of Medicine, University of Cincinnati, Cincinnati, OH, United States; ^4^Division of Rheumatology, Cincinnati Children's Hospital Medical Center, Cincinnati, OH, United States

**Keywords:** lupus nephritis, urine, biomarker, stability, SLE

## Abstract

**Background:**

The Renal Activity Index for Lupus (RAIL) is a composite score of six urinary biomarkers (neutrophil gelatinase–associated lipocalin (NGAL), monocyte chemoattractant protein-1 (MCP-1), kidney injury molecule-1 (KIM-1), ceruloplasmin, adiponectin, and hemopexin) used to monitor lupus nephritis activity in children. We tested stability of RAIL biomarkers prior to meaningful clinical use.

**Methods:**

Urine samples were tested by ELISA under shipping conditions, freeze/thaw, ambient and longer-term storage. Statistical analysis was performed *via* Deming Regression, Bland-Altman and Spearman Correlation Coefficient.

**Results:**

Biomarker concentration were comparable to freshly collected urine following storage at −80 °C for up to 3 months, and at 4 or 25 °C up to 48 h followed by −80 °C. Neither shipping on dry or wet ice exposure nor addition of two freeze-thaw cycles led to loss of signal, with excellent Spearman Correlation coefficients under all conditions.

**Conclusions:**

RAIL biomarkers are stable following short-term storage at clinically relevant conditions.

## Introduction

Lupus nephritis (LN) confers a poor prognosis, as 5–25% of patients with proliferative LN develop kidney disease within 5 years of diagnosis, and about 10% of all patients with LN will develop end-stage kidney disease (1, 2). Disease with onset in childhood or adolescence is termed childhood onset SLE, or cSLE. Outcomes for cSLE are worse than that of adults and children have higher occurrence of LN (3–5). Current laboratory testing is inadequate to diagnose LN activity effectively and evaluate response to therapy, which makes evaluating potential new therapies challenging (6, 7). In fact, traditional laboratory measures will classify patients incorrectly 30–40% of the time when compared to kidney biopsy. As such, complement levels and anti-dsDNA antibody titers are not effective for predicting the course of LN (6, 8, 9). Therefore, research has focused on developing new laboratory tests to non-invasively, accurately, and rapidly diagnose LN and detect its response to treatment.

We have previously described and validated a panel of six urinary biomarkers, neutrophil gelatinase-associated lipocalin (NGAL), monocyte chemoattractant protein-1 (MCP-1/CCL2), kidney injury molecule-1 (KIM-1), ceruloplasmin, adiponectin, and hemopexin. Considering the urinary concentration of all six biomarkers, the pediatric Renal Activity Index for Lupus (RAIL) can be calculated, where a higher score reflects high renal inflammation, and the score is >90% accurate in detecting LN activity histologic activity measures (10–12). The standard operating procedures for collecting and storing urine specimens to be used for RAIL biomarker measurement has yet to be established. We have previously demonstrated the short- and long-term of NGAL and KIM-1 in pediatric AKI patients. We found no significant difference between concentration measurements at baseline (immediate testing within 15 min of aliquoting), 24 h at 4 °C, or 24 h at −80 °C (13). In addition, NGAL and KIM-1 demonstrated long-term stability, without relevant degradation at long term storage, for up to 5 years. To further move the RAIL biomarkers toward clinical utility, we conducted stability testing for the remaining four urinary biomarkers that make up the RAIL score under common clinical and experimental conditions. We hypothesized that the markers would be stable under various clinically relevant conditions including shipping, temperatures, and longer storage.

## Materials and methods

### Patients and samples

Patients enrolled in the SLE Clinical and Research Database were eligible for the study (IRB CCHMC-2008-0635). Patients were recruited over a 1-month period in September 2021 from Nephrology and Rheumatology clinics. Inclusion criteria were diagnosis of SLE by EULAR/ACR 2019 criteria (14) with or without nephritis. There was no requirement based on disease activity. Exclusion criteria were diagnosis of kidney disease other than Lupus Nephritis, diagnosis of autoimmune disease besides SLE, urine volumes below 10 ml or patient unwilling to provide urine.

**Figure 1:**
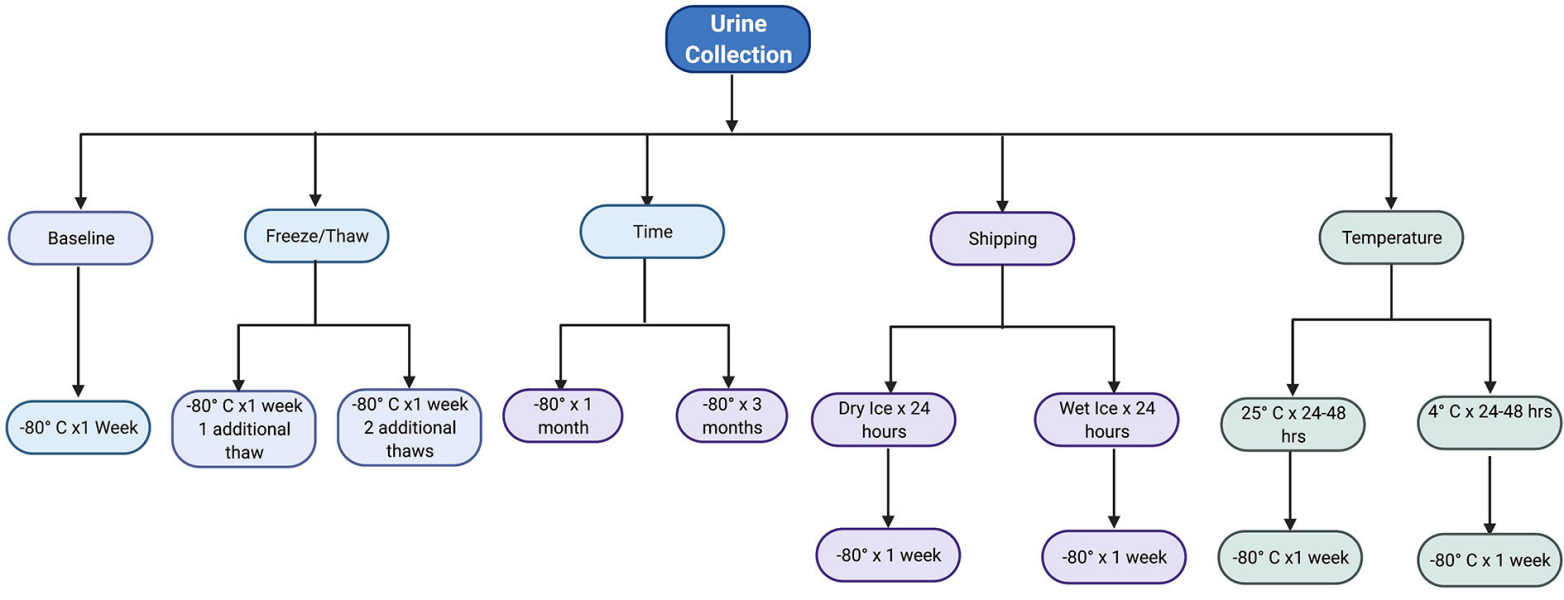
Flowchart of the 10 experimental conditions per urine sample. Image made with BioRender.

A random urine sample was collected from each patient and processed within 2 h of collection. Urine was centrifuged for 10 min at 2200x g prior to aliquoting for testing the 10 experimental conditions, as shown in Figure 1. Control condition was freezing at −80 °C and run within 1 week of collection. All thaw times were 45 min.

### Urine biomarker assays

Unless stated otherwise, biomarkers were quantified using commercial ELISA kits as per the manufacturer's instructions. MCP-1 used a four-parameter logistic curve to fit the standard curve. Adiponectin, Ceruloplasmin, and Hemopexin used a log/log curve to fit the standard curve.

Human MCP-1 was measured *via* ELISA (R&D Systems, Minneapolis, MN, DCP00), diluted 1:1, with a mean minimal detectable dose of 1.7 pg/mL. Intra-assay and inter-assay were 5% and 5.1%, respectively.

Human Adiponectin was measured by commercially available ELISA kit (R&D system, Minneapolis, MN, DRP300), diluted 1:5, with a mean minimal detectable dose of 0.246 ng/ml. Intra-assay and inter-assay coefficients of variation were 3.7 and 6.8%, respectively.

Human Ceruloplasmin was measured by ELISA Kit (Assaypro LLC, St. Charles, MO, EC4201-1), diluted 1:50, with a mean minimal detectable dose of 0.085 ng/ml. Intra-assay and inter-assay were 4.9 and 9.8%, respectively.

Human Hemopexin was measured by commercially available ELISA kit (Assaypro LLC, St. Charles, MO, EH2001-1), diluted 1:20, with a mean minimal detectable dose of 4.2 ng/ml. Intra-assay and inter-assay coefficients of variation were 4.7 and 9.2%, respectively.

### Statistical analysis

Natural log-transformation of each biomarker was performed prior to analysis to ensure normal distribution assumptions. Analyte concentrations that were above or below the limits of detection were imputed by 50% of the level of lower limit of detection (LLD) and 50% over the upper limit of detection (ULD), respectively. Means were calculated for each biomarker under each condition. Spearman correlation coefficients were calculated for each experimental condition with the control condition, with of 0.1–0.39 representing weak correlation, 0.4–0.69 representing moderate correlation, 0.7–0.89 representing strong correlation, and >0.9 representing very strong correlation (15). Conditions were then compared to baseline using Deming regression and Bland-Altman bias plots. Since the urinary biomarkers measured under different experimental conditions (including the control condition) may be subject to some degree of measurement variabilities, we took Deming Regression approach, modeling the relationship between each pair of experimental conditions with the control condition. For the Bland-Altman plots, the differences between the experimental vs. control condition for a given urinary biomarker are shown in the y-axis while the corresponding measurement under the control condition is shown in the x-axis. For each urinary biomarker the mean difference, or bias, and the 95% limits of agreement was displayed (16). When the majority of measurements were within the 95% limits of agreement, then a given biomarker was considered stable across the tested experimental conditions. For month 1 and month 3 samples, all urinary biomarkers were repeated four times, so intraclass correlation coefficients (ICC) and coefficients of variation (CV) were calculated. An ICC of <0.5 represents poor reliability, between 0.5 and 0.75 represent moderate reliability and 0.75 to 0.9 indicates good reliability, and >0.9 indicates excellent reliability (17). All statistical analyses were performed using SAS 9.4 (Cary, NC).

## Results

Patient urine specimens were obtained in a serial fashion, resulting in 10 urine samples from 10 patients. The average age of the patient population was 19.09 (± 2.51) years. There were 9 females (90%), and the majority were Caucasian (70%), with the remaining African American. Most of the patients (80%) did not have a diagnosis of lupus nephritis, and the two that did have Class II Lupus Nephritis. The urine volumes were sufficient that each collection was able to be run under each experimental collection. MCP-1 had the least number of samples below the lower limit of detection, only 1/220 (0.45%). Adiponectin had 44/220 (20%) and Hemopexin had 25/220 (11.36%) below the lower limit of detection. Ceruloplasmin has samples both above and below the limits of detection, each 44/220 (20%).

### Adiponectin

**Figure 2:**
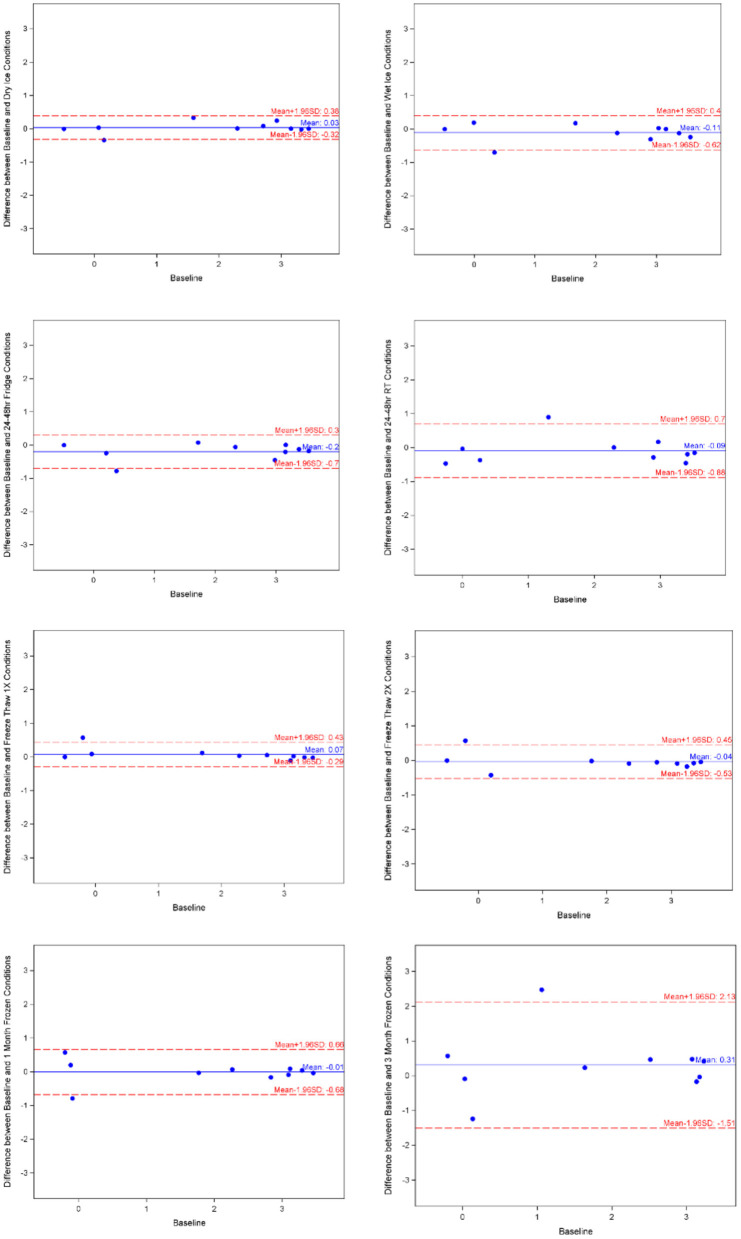
Adiponectin Bland-Altman charts by experimental condition. Image made with BioRender.

The means of adiponectin under baseline condition was 1.935 ± 1.521, with minimum of −0.486 and maximum of 3.441 from the 10 samples included. As shown in Table 1, the log-transformed means for experimental conditions ranged from 1.626 ± 1.482 (average 15.97% decrease, after 3-month storage) to 2.132 ± 1.476 (under Fridge condition). Spearman correlation coefficients showed duplicates had very good correlation within each sample run, all >0.9, except for room temperature, which was good at 0.888. The Bland-Altman plots (Figure 2) and Deming-Regression (Table 2) show that differences in means for each experimental condition are due to random error. Finally, Table 3 shows ICC for 1 month and 3 months storage conditions, revealing excellent reliability.

**Table 1:** Average urinary biomarker concentration under each experimental condition with spearman correlation coefficients.

	**Condition**	**Mean**	**St dev**	**Min**	**Max**	**Spearman correlation coefficient**	***P*** **-value**
Adiponectin	Baseline	1.935	1.521	−0.486	3.441	0.973	<0.01
	Dry ice	1.901	1.466	−0.486	3.438	0.979	<0.01
	Wet ice	2.045	1.530	−0.486	3.681	0.961	<0.01
	Fridge	2.132	1.476	−0.486	3.626	1.00	<0.01
	RT	2.025	1.530	−0.018	3.615	0.888	<0.01
	FT1	1.862	1.627	−0.486	3.469	0.998	<0.01
	FT2	1.976	1.596	−0.486	3.483	0.988	<0.01
	1MO	1.950	1.532	−0.486	3.478	0.982	<0.01
	3MO	1.626	1.482	−0.486	3.220	0.910	<0.01
Ceruloplasmin	Baseline	3.618	2.248	0.754	6.669	0.991	<0.01
	Dry-ice	3.552	2.180	0.754	6.669	1.00	<0.01
	Wet ice	3.553	2.305	0.754	6.669	1.00	<0.01
	Fridge	3.483	2.250	0.754	6.669	0.984	<0.01
	RT	3.614	2.175	0.754	6.669	1.00	<0.01
	FT1	3.692	2.310	0.754	6.669	0.869	<0.01
	FT2	3.491	2.326	0.754	6.669	1.00	<0.01
	1MO	3.803	2.071	0.754	6.669	1.00	<0.01
	3MO	4.245	1.538	2.259	6.669	0.962	<0.01
Hemopexin	Baseline	5.522	0.835	3.738	6.575	0.915	<0.01
	Dry ice	5.524	0.887	3.738	6.643	0.989	<0.01
	Wet ice	5.530	0.847	3.738	6.663	0.976	<0.01
	Fridge	5.510	0.862	3.738	6.741	0.964	<0.01
	RT	5.413	1.004	3.738	6.663	0.963	<0.01
	FT1	5.488	0.935	3.738	6.602	0.961	<0.01
	FT2	5.470	0.898	3.738	6.533	0.967	<0.01
	1MO	5.536	0.905	3.738	6.712	0.973	<0.01
	3MO	5.817	0.897	4.187	6.897	0.952	<0.01
MCP-1	Baseline	4.558	1.600	1.760	6.448	1.00	<0.01
	Dry ice	4.596	1.562	2.255	6.379	1.00	<0.01
	Wet ice	4.556	1.633	1.888	6.468	1.00	<0.01
	Fridge	4.503	1.710	1.386	6.397	1.00	<0.01
	RT	4.426	1.813	0.987	6.423	0.999	<0.01
	FT1	4.553	1.582	2.135	6.376	0.993	<0.01
	FT2	4.471	1.751	1.456	6.408	1.00	<0.01
	1MO	4.673	1.504	2.309	6.402	0.992	<0.01
	3MO	4.607	1.601	2.156	6.467	1.00	<0.01

**Table 2:** Deming-Regression Intercept and beta-coefficient for each biomarker under each experimental condition.

	**Dry ice**	**Wet ice**	**Fridge**	**Room temperature**	**Freeze thaw 1X**	**Freeze thaw 2X**	**1 month frozen**	**3 month frozen**
Adiponectin	0.04 (95% CL −0.24, 0.32)	0.1 (95% CL −0.44, 0.64)	0.26 (95% CL −0.28, 0.79)	0.08 (95% CL −0.56, 0.72)	−0.21 (95% CL −0.64, 0.22)	−0.06 (95% CL −0.67, 0.56)	0 (95% CL −0.96, 0.96)	−0.24 (95% CL −2.09, 1.62)
	0.96 (95% CL 0.87, 1.06)	1.01 (95% CL 0.83, 1.18)	0.97 (95% CL 0.79, 1.15)	1.01 (95% CL 0.8, 1.22)	1.07 (95% CL 0.92, 1.22)	1.05 (95% CL 0.84, 1.26)	1.01 (95% CL 0.68, 1.34)	0.96 (95% CL 0.34, 1.59)
Ceruloplasmin	0.05 (95% CL −0.53, 0.63)	−0.16 (95% CL −0.45, 0.13)	−0.14 (95% CL −0.44, 0.16)	0.11 (95% CL −0.41, 0.64)	−0.03 (95% CL −0.36, 0.3)	−0.25 (95% CL −0.66, 0.15)	0.56 (95% CL −2.2, 3.31)	1.85 (95% CL 0.79, 2.92)
	0.97 (95% CL 0.86, 1.08)	1.03 (95% CL 0.97, 1.08)	1 (95% CL 0.93, 1.07)	0.97 (95% CL 0.87, 1.06)	1.03 (95% CL 0.93, 1.12)	1.04 (95% CL 0.96, 1.11)	0.9 (95% CL 0.38, 1.41)	0.66 (95% CL 0.43, 0.89)
Hemopexin	−0.35 (95% CL −1.4, 0.71)	−0.07 (95% CL −0.5, 0.36)	−0.19 (95% CL −1.17, 0.8)	−1.39 (95% CL −6.45, 3.68)	−0.74 (95% CL −3.56, 2.08)	−0.49 (95% CL −2.68, 1.69)	−0.46 (95% CL −2, 1.07)	−0.22 (95% CL −6.46, 6.02)
	1.06 (95% CL 0.88, 1.24)	1.01 (95% CL 0.93, 1.1)	1.03 (95% CL 0.84, 1.22)	1.23 (95% CL 0.38, 2.09)	1.13 (95% CL 0.65, 1.6)	1.08 (95% CL 0.72, 1.44)	1.09 (95% CL 0.81, 1.36)	1.09 (95% CL 0.04, 2.15)
MCP-1	0.15 (95% CL −0.85, 1.14)	−0.1 (95% CL −0.62, 0.42)	−0.37 (95% CL −0.86, 0.12)	−0.75 (95% CL −1.53, 0.04)	0.05 (95% CL −0.85, 0.94)	−0.52 (95% CL −0.75, −0.29)	0.39 (95% CL −0.37, 1.15)	0.04 (95% CL −0.87, 0.96)
	0.98 (95% CL 0.79, 1.16)	1.02 (95% CL 0.92, 1.12)	1.07 (95% CL 0.98, 1.16)	1.13 (95% CL 0.99, 1.28)	0.99 (95% CL 0.82, 1.15)	1.09 (95% CL 1.04, 1.15)	0.94 (95% CL 0.8, 1.08)	1 (95% CL 0.83, 1.17)

**Table 3:** ICC values at 1- and 3-months of storage.

**1 month**	**ICC**	**Lower bound**	**Upper bound**
Adiponectin	0.997	0.974	0.994
Ceruloplasmin	0.998	0.996	0.999
Hemopexin	0.994	0.987	0.997
MCP-1	0.998	0.996	0.999
**3 month**	**ICC**	**Lower bound**	**Upper bound**
Adiponectin	0.908	0.819	0.954
Ceruloplasmin	0.991	0.981	0.996
Hemopexin	0.949	0.898	0.975
MCP-1	0.998	0.996	0.999

### Ceruloplasmin

**Figure 3:**
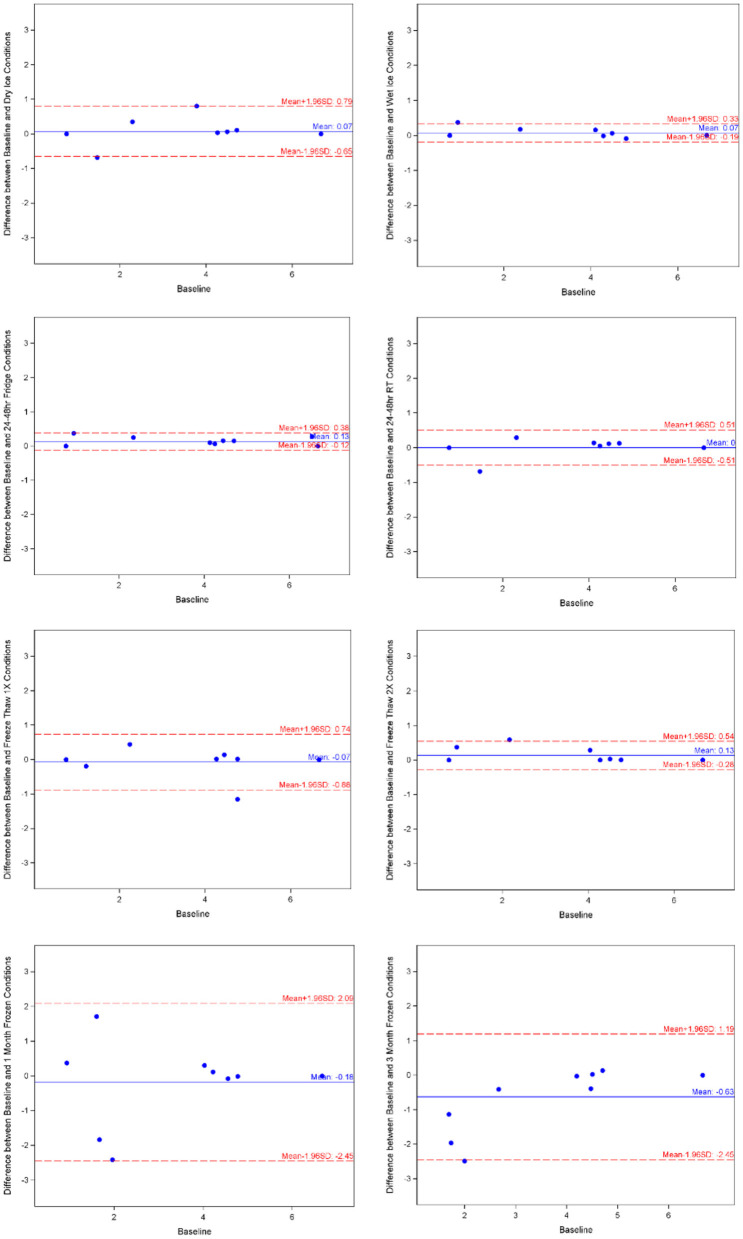
Ceruloplasmin Bland-Altman charts by experimental condition. Image made with BioRender.

Likewise, mean for ceruloplasmin under baseline condition was 3.618 ± 2.248, with minimum of 0.754 and maximum of 6.669 from the 10 samples. As shown in Table 1, the log-transformed means for experimental conditions ranged from 3.483 ± 2.25 (average 3.73% decrease, under Fridge condition) to 4.245 ± 1.538 (after 3-month storage). Spearman correlation coefficients showed duplicates had very good correlation within each sample run, all >0.9 apart from the first freeze thaw experiment. The Bland-Altman plots and Deming-Regression are shown in Figure 3 and Table 2 respectively. There was some variability within the lower limits of detection but overall, differences in means for each experimental condition are due to random error. Finally, Table 3 shows ICC for 1 month and 3 months storage conditions, revealing excellent reliability.

### Hemopexin

**Figure 4:**
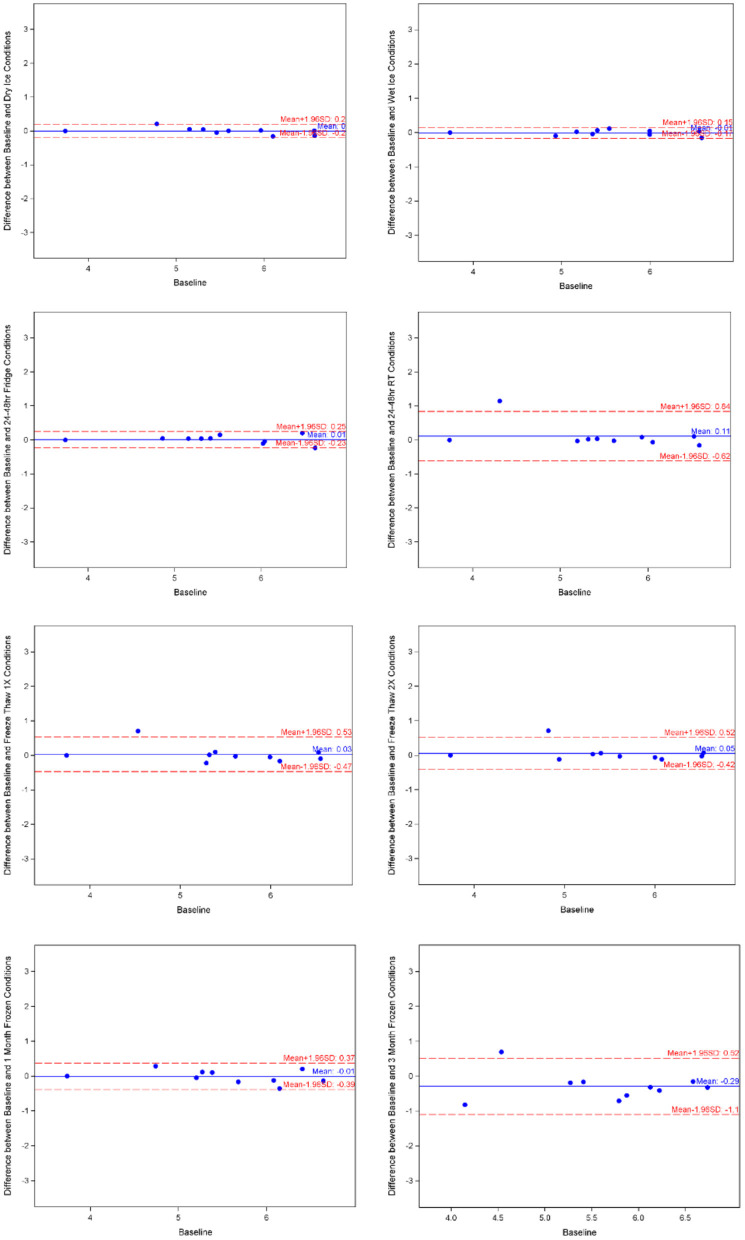
Hemopexin Bland-Altman charts by experimental condition. Image made with BioRender.

Mean for hemopexin under baseline condition was 5.522 ± 0.835, with minimum of 3.738 and maximum of 6.575 from the 10 samples. As shown in Table 1, the log-transformed means for experimental conditions ranged from 5.413 ± 1.004 (average 1.8% decrease, under Room Temperature) to 5.817 ± 0.897 (after 3-month storage). Spearman correlation coefficients showed duplicates had very good correlation within each sample run, all >0.9. The Bland-Altman plots (Figure 4) and Deming-Regression (Table 2) show that differences in means for each experimental condition are due to random error. Finally, Table 3 shows ICC for 1 month and 3 months storage conditions, revealing excellent reliability.

### MCP-1

**Figure 5:**
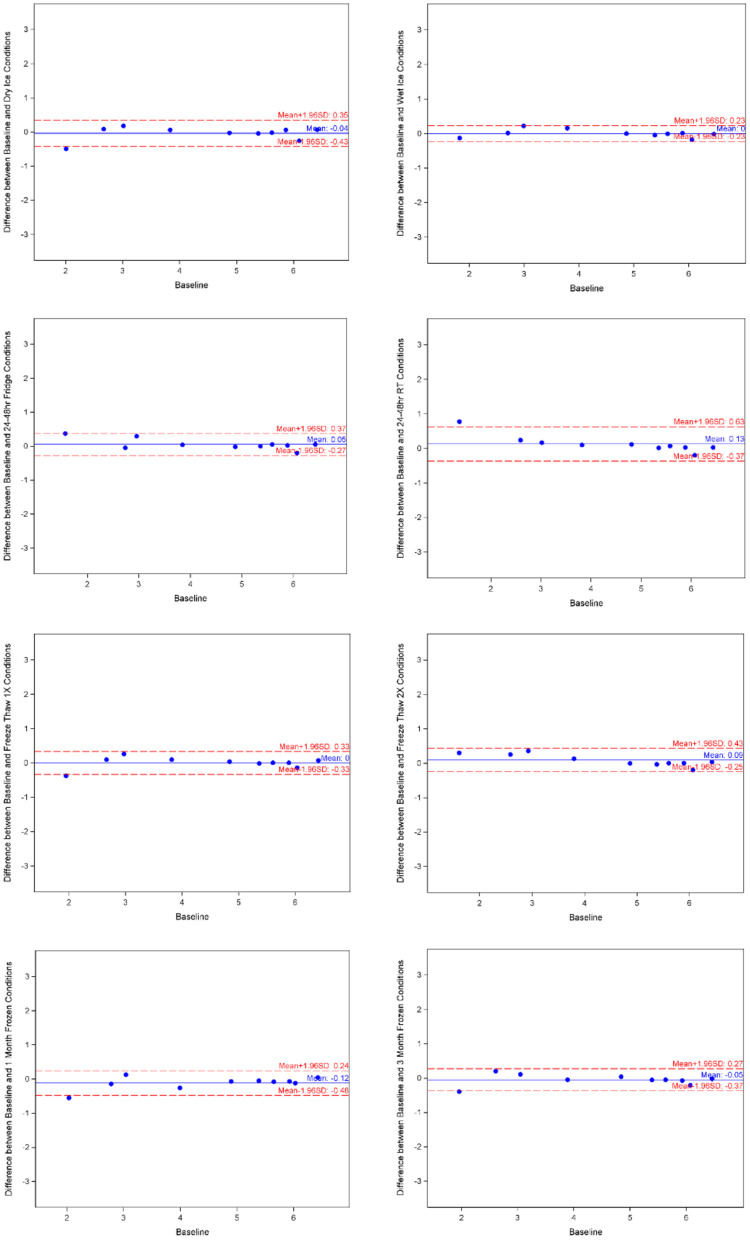
MCP-1 Bland-Altman charts by experimental condition. Image made with BioRender.

Mean of MCP-1 was 4.558 ± 1.600, with minimum of 1.760 and maximum of 6.448 from the 10 samples at baseline condition. As shown in Table 1, the log-transformed means for experimental conditions ranged from 4.426 ± 1.813 (average 2.90% decrease, under Room Temperature) to 4.673 ± 1.504 (after 1-month storage). Spearman correlation coefficients showed duplicates had very good correlation within each sample run, all >0.9. The Bland-Altman plots (Figure 5) and Deming-Regression (Table 2) show that differences in means for each experimental condition are due to random error. Finally, Table 3 shows ICC for 1 month and 3 months storage conditions, revealing excellent reliability.

## Discussion

In this study, we meaningfully add to the growing literature on using urinary biomarkers for LN evaluation. Exploration of stability is important in anticipation to move the RAIL into the clinical realm and consider use of the RAIL biomarkers in clinical trials.

We have previously shown the stability of NGAL and KIM-1 under different temperatures, freeze/thaw conditions and long-term storage (13). We now add four additional biomarkers of importance to lupus nephritis research: MCP-1, Adiponectin, Hemopexin and Ceruloplasmin. We tested effect of temperature, freeze/thaw, shipping, and storage for months, without significant differences between baseline and the experimental conditions. Importantly, we provide new data on the stability of urinary adiponectin, hemopexin, and ceruloplasmin that has not previously been reported.

We showed that the four other biomarkers included in the RAIL are stable at room temperature and 4 °C for up to 48 h. This has been replicated for other biomarkers as well. Wang et al. (18) evaluated effect of various temperature (4 °C, 22 °C, 40 °C) and short-term storage (24 h, 48 h) on the metabolite signature in urine. The control used was −20 °C. As in this study, they also found stability of the metabolite signature at 4 °C for 24 and 48 h, as well 22 °C for 24 h, however not 48 h. There was no integrity of the metabolite signature at 40 °C for any length of time. The effect of temperature on urinary KIM-1 and NGAL have been studied in various other studies (19, 20). Both proteins have been shown to be stable at 4 °C at 48 h as well as 25 °C at 48 h. NGAL has further been shown to be stable for up to 7 days at room temperature, 4 °C and −20 °C, which is not the case with KIM-1 (21, 22). Chang et al. (20), besides studying KIM-1 and NGAL, as well as other biomarkers, also evaluated MCP-1, and has shown it to be stable after 48 h at 4 °C, 48 h at 25 °C, no centrifugation but immediate aliquot and storage at −80 °C.

The data on longer-term storage are somewhat conflicting. Pennemans et al. (23) evaluated KIM-1 and showed good recovery at −80 °C for up to 1.5 years, but a bigger decline in biomarker concentration with storage at −20 °C. De Vrie et al. (22) evaluated KIM-1 and NGAL at −80 °C for 6 months, with both markers showing stability. Herrington et al. (24) showed urine albumin preservation for up to 6 months at −80 °C or −40 °C, but not −20 °C. In contrast, several authors have shown more variability in biomarker stability, even at −80 °C. Nauta et al. (25) evaluated multiple markers, including KIM-1, NGAL at 1 year in −20 °C or −80 °C. All markers except urine cystatin C showed a gradual decline in concentration, which seemed to stabilize after 6 months. Liu et al. (26) compared storage at −70 °C. KIM-1, NGAL, IL-18, liver-type fatty acid-binding protein (L-FABP) in hospitalized and non-hospitalized patients, where processing could be up to 6 h. Median duration of storage was 17.8 months for inpatient, and 14.6 months for outpatient samples. For NGAL, IL-18, L-FABP, storage time was not significantly associated with biomarker levels. KIM-1 levels were lower with longer storage times in the outpatient group, but this was finding was not found with the inpatient group.

The effect of freeze thaw cycles was studied for urine NGAL and KIM-1 previously. We newly evaluated the effects of freeze thaws on hemopexin, adiponectin, MCP-1 and ceruloplasmin concentrations. Hogan et al. (21) showed that NGAL was stable for up to 3 freeze/thaw cycles at −20 °C. Further, Herrington et al. (24) showed that urine albumin is preserved after 3 freeze/thaw cycles. These reports are similar to this study, with storage temperature of −80 °C. Pennemans et al. (23) performed a study in KIM-1, comparing the difference between 3 h and 24-h thaw period compared to 1 h, with optimal thawing time being 1 h.

Our study is the first to show the stability of biomarkers under study shipping conditions, both under dry ice and with wet ice. This has important implications for the measurement of these biomarkers by select clinical laboratories in stored urine samples from patients and from previous clinical trials.

One limitation with this study may be the small sample size, including of patients with lupus nephritis, which may reduce its generalizability. While most of the biomarkers were within the detectable range of the assay kits, ceruloplasmin had percentages of samples both above and below outside range of detection on assay. However, the amount below the detectable limit was reasonable, and the amount above detection that did not show large amount of decay.

In conclusion, our study demonstrates stability of hemopexin, ceruloplasmin, MCP-1 and adiponectin under different conditions that are important for clinical and experimental use, including up 48 h at room temperature, 4 °C, as well as up to 3 months at −80 °C storage. Importantly, we provide new data that these biomarkers are stable under storage with both wet and dry ice. With rising use of biomarkers for both clinical use and research, these results are reassuring that shipping, as well as long term storage and multiple freeze/thaw cycles can be employed prior to batch measurement.

## Data availability statement

The original contributions presented in the study are included in the article/supplementary material, further inquiries can be directed to the corresponding author.

## Ethics statement

The studies involving human participants were reviewed and approved by Cincinnati Children's Hospital IRB (IRB CCHMC-2008-0635). Written informed consent to participate in this study was provided by the participants' legal guardian/next of kin.

## Author contributions

Research idea and study design: EC, HB, and PD. Data acquisition: JR. Data analysis and interpretation: BH, EC, HB, and PD. Statistical analysis: BH and TQ. Supervision or mentorship: HB and PD. All authors contributed to the article and approved the submitted version.

## Funding

This study was supported by the Lupus Foundation of America, Falk Foundation, and Portico (P30AR076316). PD was supported by a grant from the NIH (P50DK096418).

## Conflict of interest

Author PD is a co-inventor on submitted patents for the use of NGAL as a biomarker of kidney disease. The remaining authors declare that the research was conducted in the absence of any commercial or financial relationships that could be construed as a potential conflict of interest.

## Publisher's note

All claims expressed in this article are solely those of the authors and do not necessarily represent those of their affiliated organizations, or those of the publisher, the editors and the reviewers. Any product that may be evaluated in this article, or claim that may be made by its manufacturer, is not guaranteed or endorsed by the publisher.
